# Susceptibility of BS90 *Biomphalaria glabrata* snails to infection by *Sm*LE *Schistosoma mansoni* segregates as a dominant allele in a cluster of polymorphic genes for single-pass transmembrane proteins

**DOI:** 10.1371/journal.pntd.0012474

**Published:** 2024-09-16

**Authors:** Michael S. Blouin, Stephanie R. Bollmann, Winka Le Clec’h, Frédéric D. Chevalier, Timothy J. C. Anderson, Jacob A. Tennessen

**Affiliations:** 1 Department of Integrative Biology, Oregon State University, Corvallis, Oregon, United States of America; 2 Host Parasite Interaction Program, Texas Biomedical Research Institute, San Antonio, Texas, United States of America; 3 Disease Intervention and Prevention Program, Texas Biomedical Research Institute, San Antonio, Texas, United States of America; 4 Immunology and Infectious Diseases, Harvard T.H. Chan School of Public Health, Boston, Massachusetts, United States of America; Université de Perpignan: Universite de Perpignan Via Domitia, FRANCE

## Abstract

The trematodes that cause schistosomiasis in humans require aquatic snails as intermediate hosts. Identifying the genes in snails at which allelic variation controls resistance to infection by schistosomes could lead to novel ways to break the cycle of transmission. We therefore mapped genetic variation within the BS90 population of *Biomphalaria glabrata* snails that controls their resistance to infection by the SmLE population of *Schistosoma mansoni*. A marker in the *PTC2* genomic region strongly associates with variation in resistance. The *S*-haplotype, which confers increased susceptibility, appears to be almost completely dominant to the *R*-haplotype, which confers increased resistance. This result suggests a model in which the parasite must match a molecule on the host side to successfully infect. The genomic region surrounding our marker shows high structural and sequence variability between haplotypes. It is also highly enriched for genes that code for single-pass transmembrane (TM1) genes. Several of the TM1 genes present on the *S*-haplotype lack orthologs on the *R*-haplotype, which makes them intriguing candidate genes in a model of dominant susceptibility. These results add to a growing body of work that suggests TM1 genes, especially those in this exceptionally diverse genomic region, may play an important role in snail-schistosome compatibility polymorphisms.

## Author summary

Schistosomiasis is a parasitic disease of humans that is caused by trematodes in the genus *Schistosoma*. Schistosomes require aquatic snails as an intermediate host. Infected snails shed parasites into water, which then infect people through their skin. *Biomphalaria glabrata* host snails show substantial genetic variation for susceptibility to infection by *Schistosoma mansoni*. Via genome-wide mapping we identified the general location of a gene in the *B*. *glabrata* genome at which different alleles control whether snails can be infected or not. The allele that causes susceptibility is dominant to the allele that causes resistance. This uncommon pattern suggests that an invading parasite must match some target on the host side to successfully establish an infection. The mapped snail genomic region contains multiple genes that code for proteins that span cell membranes. These proteins have an extracellular piece that can interact with molecules outside of cells. We hypothesize that these proteins are the targets of molecules produced by the schistosome. If so, then identifying the interacting molecules on the schistosome side, and how they interact with snail proteins, might reveal ways we could genetically modify snails to make them unable to transmit schistosomes to humans.

## Introduction

Schistosomes are trematode parasites that use humans as definitive hosts and require aquatic snails as intermediate hosts. Schistosomiasis affects over 200 million worldwide, causing severe and chronic illness [1]. Among parasitic diseases, schistosomiasis is second only to malaria in public health impact [2]. No effective vaccines exist. Mass drug administration to humans is less effective for reducing transmission than controlling the intermediate host [3]. However, traditional means of snail control, such as widespread application of molluscicides, are problematic [4,5]. New approaches to stopping transmission at the snail stage are needed. Identifying key genes and molecular pathways in snails that control heritable variation in resistance to schistosomes is an important first step.

Schistosome eggs are shed in human feces or urine. Upon contact with fresh water, they hatch into miracidia that attempt to infect snails. They then clonally produce thousands of cercariae that exit the snail in search of another human host. Snails are the obligate intermediate host for most parasitic trematodes, and the two have been coevolving in an evolutionary arms race for millions of years [6]. There is substantial interaction for compatibility among populations of snails and schistosomes, such that populations of snails can be highly resistant to certain populations of schistosome, and highly susceptible to others, while populations of schistosome can be highly infectious to some populations of snails, but not to others [7–11].

So, there must be substantial genetic variation on both sides. Understanding how the network of interacting genes works to control compatibility between snails and schistosomes could reveal new targets for potentially blocking transmission. In particular, it is possible that this information could be used to genetically alter natural snail populations to make them less able to transmit the parasite [12–15].

*Biomphalaria glabrata* is the most important intermediate host for transmission of *Schistosoma mansoni* in the Americas. What is known about molecular interactions between *B*. *glabrata* and *S*. *mansoni* has been reviewed in [16–19]. Genome-wide mapping studies using various populations of *B*. *glabrata* and *S*. *mansoni* have revealed five genomic regions in which allelic variation in snails controls resistance to *S*. *mansoni* [20–24]. Likely causal genes are known for two of those regions: *grctm6*, which codes for a single-pass transmembrane protein that is probably involved in pathogen recognition [25–27], and *Cu-Zn SOD*, which is involved in the oxidative burst [21,28].

BS90 is a Brazilian population of *B*. *glabrata* that is highly resistant to infection by most of the common laboratory populations of *S*. *mansoni* that have been tested against it. BS90 was reportedly isolated in Salvador, Bahia, Brazil, in the 1960s, and has been used in laboratories in the US since the 1990s ([29], cited in [20]; C. Bayne, 2006, pers. comm.). BS90 snails have been distributed for years by the Biomedical Research Institute’s Schistosomiasis Resource Center (https://www.afbr-bri.org/schistosomiasis/) as the exemplar resistant population of *B*. *glabrata*, so are in use in many laboratories. There have been dozens of functional and genetic studies using BS90 snails on why they are so much more resistant than other commonly-used laboratory populations of *B*. *glabrata* [30–35].

Théron et al. [10] showed that the SmLE population of *S*. *mansoni* could infect about half of the individuals in their population of BS90. In their study, dose-response curves plateaued at about 50%. This result suggests that about half the snails are highly resistant to SmLE, a result consistent with heritable variation for resistance in that BS90 population (e.g. same pattern as observed by Théron et al. [36] and Tennessen et al. [22] for *B*. *glabrata* and *S*. *mansoni* from Guadeloupe). So, our goal was to map the genomic location/s of any loci behind that putative heritable variation in BS90.

## Methods

### Ethics statement

The Oregon State University Institutional Animal Care and Use Committee approved this research under Animal Care and Use Protocols 2021–0213 and 5115.

### Study populations

For this study we first used tissue samples from BS90 snails that had been challenged by *S*. *mansoni* in T. Anderson’s laboratory at the Texas Biomedical Research Institute as part of a different experiment. They did a QTL-mapping experiment to map *S*. *mansoni* genes involved in the difference between the SmLE and SmBRE populations of *S*. *mansoni* in their ability to infect BS90 snails (SmBRE cannot infect BS90 snails, while SmLE can infect half of them). Each snail had been challenged with 10 miracidia that were F2s from an SmLE x SmBRE cross, which resulted in ~38% of the snails getting infected. Each snail was scored as infected or not uninfected by scoring whether it shed cercariae between weeks 4–10 after challenge. Of these snails, we used 96 infected and 96 uninfected snails as cases and controls in a preliminary genome-wide association study (GWAS) at Oregon State University to identify any genomic regions that might be involved in the variation in resistance.

Subsequent experiments were conducted in Blouin’s laboratory at OSU using a population of SmLE obtained from Anderson’s laboratory in 2019, and a population of BS90 obtained from Anderson’s laboratory in 2017. We also used a population of BS90 that we subjected to one generation of selection for resistance to SmLE in Blouin’s laboratory (hereafter, the ‘BS90-Sel1’ population). BS90-Sel1 typically shows ~11% fewer infected snails than the base BS90 population in side-by-side challenges with SmLE (S1 Data). The BS90 base population has always been maintained in the absence of any parasite selection pressure. Anderson’s laboratory originally obtained their SmLE in 2013 from Phillip Loverde’s laboratory at the University of Texas Health Science Center, San Antonio (now UT Health, San Antonio), and their population of BS90 snails from the Schistosomiasis Resource Center in 2013.

### Husbandry and schistosome challenges at OSU

At OSU, snails were housed in 7.5 liter plastic tanks containing artificial spring water [37], and fed green-leaf lettuce. We challenged 6 to 9 mm diameter snails individually in 24-well plates (in ~1 ml water) using 5, 10 or 20 miracidia of SmLE-population *S*. *mansoni* per snail. Snails were kept in darkened tubs (~24 per tub) for four weeks, and then checked every week or two for shedding, for six more weeks. Snails that did not shed within the 10-week window were classified as uninfected. Individuals that died without shedding before 10 weeks were not included in the analysis.

SmLE was maintained at OSU by passaging through laboratory mice (*Mus musculus*) or hamsters (*Mesocricetus auratus*) as the mammalian host, and through M-line population *B*. *glabrata*, which are highly susceptible to SmLE (M-line ‘MT0’ snails were obtained from the Schistosomiasis Resource Center in 2018).

### Genome wide association study

We used 96 infected snails and 96 uninfected snails as cases and controls in a PoolSeq GWAS [38]. DNA was extracted from head foot tissue by CTAB extraction [39] and quantified by Qubit fluorometric assay (Thermo Fisher). We pooled the DNA from each of 12 snails in equal DNA concentrations to create each subpool. We created 16 independent subpools of 12 snails each. Eight subpools were created using infected snails, and eight were created using uninfected snails. We independently barcoded each subpool and combined the 16 libraries (equimolar concentrations, verified by qPCR) for Illumina sequencing. After sequencing, we combined reads from the eight infected-snail subpools to create a single ‘infected’ pool, and combined reads from the eight uninfected-snail subpools to create a single ‘uninfected’ pool (S1 Fig part A). We then compared these two main pools across the entire genome via sliding-window *F*_st_ analysis as in Tennessen et al. (2020 [24]) (10 Kb windows, moving 5 Kb each interval). We aligned our reads to PacBio genome assemblies created from four different inbred lines of BS90 snails: the iBS90 assembly from Bu et al. (2022 [35]), and assemblies from three different inbred lines from Blouin’s laboratory, which are named FSS5, FRS11 and F6RR (all independently derived from their outbred population of BS90 via 2–3 generations of selfing).

Sliding window *F*_st_ analysis with small sample sizes tend to yield ‘peaky’ plots, with random peaks that appear to stand out from the background level of *F*_st_. To determine which peaks are likely to be ‘real’, we estimated the maximum height of random peaks that appeared when we compared artificial pools that each contained an equal mix of infected and uninfected subpools (i.e. we mixed and matched the 16 barcoded libraries to create artificial groups of 8 subpools each, each of which contained reads from four subpools of infected snails and four from uninfected snails; see S1 Fig Part A) We found no random peaks greater than *F*_st_ ~0.06 when comparing our artificial groups (S1 Fig Part B). So, we consider only peaks substantially larger than this value to be interesting.

We initially aligned our Illumina reads to the FSS5 BS90 inbred line genome from Blouin et al. (2022) [40], and to the iBS90 inbred line genome from Bu et al. (2022 [35]). This analysis identified a ~ 7–8 Mb region on linkage group 16 (LG16) that contains the *PTC2* region identified by Tennessen et al. (2020 [24]) (see Results below). This region is structurally very complex, with many large insertions, deletions and regions of low sequence identity that span multiple megabases [24,40]. Given the structural complexity of the region, results could depend on which reference sequence was used to align the Illumina reads (i.e. given large indels and regions of low sequence identity, Illumina reads might align to some assemblies but not others). There are two haplotypes at the *PTC2* in BS90 snails, which we call *R* and *S* (see Results below). Bu et al.’s [35] assembly and the FSS5 assembly are both from snails that were *SS* at the *PTC2* marker. Therefore, we also aligned our reads to PacBio assemblies from two additional inbred lines, F6RR and FRS11, which are both genotype *RR* at the *PTC2*. Hereafter we will refer to the four inbred lines as iBS90(SS), FSS5(SS), F6RR(RR) and FRS11(RR) for ease of remembering which assemblies carry the *R* vs. *S* haplotype.

### Validation of PTC2 association using independent samples of outbred snails

To independently validate the GWAS result, we used samples from another experiment in which BS90 and BS90-Sel1 snails had been challenged using either 5, 10 or 20 miracidia of SmLE. We genotyped all these snails using a PCR marker in the *PTC2* region that distinguished between the *R* and *S* haplotypes via an agarose gel screen (genomic location, primers and PCR conditions in S1 Table). For each snail population (BS90 or BS90-Sel1), we combined the three miracidial dose groups together. We justified pooling because the pattern of association between percentage infected and genotype was very similar among the three miracidial doses. We used binary logistic regression in Systat 13.2 to test the population effect (BS90 vs BS90-Sel1) and to test the effect of substituting one allele for another on infection risk under a model of complete dominance.

The sliding-window *F*_st_ analyses showed two adjacent peaks, one of which contains the *PTC2* region, with a trough between them. We therefore also genotyped the validation snails at a marker at the edge of the trough and a marker in the second peak to test whether there was evidence for a second causal locus (primers and locations in S1 Table).

### Illumina and PacBio sequencing, and bioinformatics processing

Illumina sequencing libraries were created with the TruSeq DNA LT kit. Library preparation, pooling and sequencing were done at OSU’s Center for Quantitative Life Sciences (CQLS) core facility. The 16 barcoded libraries for the PoolSeq GWAS were combined and sequenced via paired-end reads to an average depth of 27.6 reads per pool, using an Illumina HiSeq 3000 (Illumina data available at NCBI, SRA BioProject Accession PRJNA1106909).

To prevent generating false sequence variants correlated with resistance, we filtered out reads of *S*. *mansoni* origin that are expected to be found in infected snail pools. Reads were converted to FASTA format, and BLASTN (version 2.6.0) was used to identify reads matching the *S*. *mansoni* reference genome, (v. 5.2, [41] with an E-value cutoff of 1e-040. These reads (and their mate pairs) were filtered out before downstream analysis. Filtered FASTQ files had Illumina adaptors removed with Cutadapt (v. 1.15, [42]), and were trimmed with Trimmomatic (v. 0.30, [43]) before alignment to the different BS90 genomes with BWA-MEM (v. 0.7.12 [44]). Pileup files were generated using SamTools version 1.3 [45], and variants for *F*_st_ analysis were filtered for a minimum mean allele frequency of 0.05 and a minimum mean depth of 5. Overlapping windows of 10 kb with at least 150 SNPs were used for visualization of *F*_st_ and heterozygosity (expected heterozygosity per segregating site, calculated using all the samples together).

The PacBio assembly from FSS5(SS) is available from Blouin et al. (2022 [40]), and the iBS90(SS) assembly from Bu et al. (2022 [35]). The PacBio assemblies for F6RR(RR) and FRS11(RR) were each sequenced from the genomic DNA of a single adult inbred snail. Pacbio libraries were made with the standard Sequel SMRTbell template kit. F6RR(RR) was sequenced on a Sequel I and assembled with HGAP4/falcon [46,47]. FRS11(RR) was sequenced on a Sequel II and assembled with Flye [48,49]. PacBio assemblies for F6RR(RR) and FRS11(RR) are available at NCBI BioProject Accession PRJNA1106909.

Using BLASTN and filtering for longest matches, we used the iBS90(SS) assembly and linkage map [35] to scaffold and order contigs from FSS5(SS), F6RR(RR), and FRS11(RR) along the 20 Mb of iBS90 contig 17 (JAKZJK010000017.1), which contains the *PTC2* region. iBS90(SS) and FRS11(RR) are the two best assemblies, so we use them in the figures below.

## Results

### GWAS

**Fig 1:**
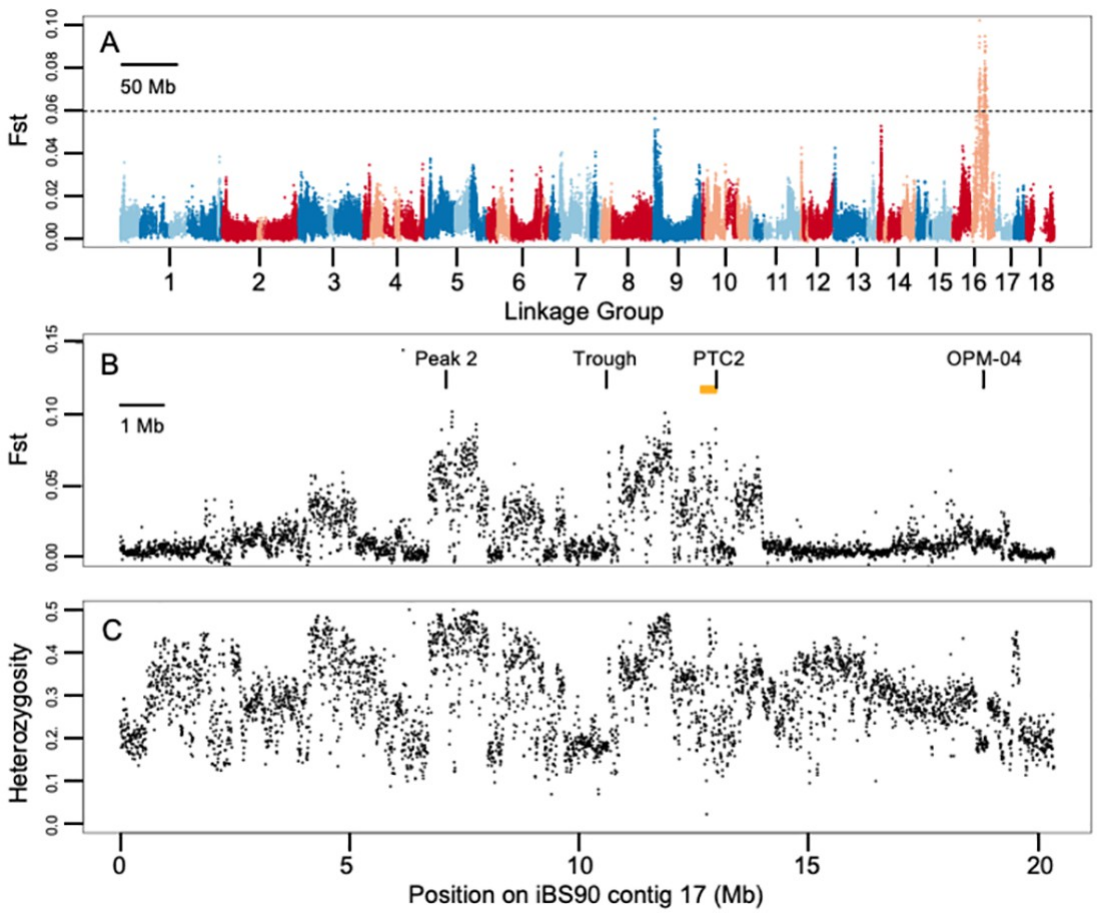
**(A) Sliding window *F***_**st**_ **analysis (10 kb windows) across entire genome, for reads aligned to the iBS90 PacBio assembly**. Linkage group (LG) numbers follow [35]. Alternating red and blue indicate LGs; alternate light and dark colors indicate contigs within each LG. Only the peak on LG16 (contig 17) is substantially higher than the *F*_st_ ~ 0.06 cutoff indicated by the dotted line (estimated from S1 Fig Part B). (B) Closeup of *F*_st_ across the 20 Mb region encompassed by iBS90 contig 17 (JAKZJK010000017.1). Orange bar = approximate location of original *PTC2* region identified by Tennessen et al. (2020 [24]). “Peak 2”, “Trough” and “PTC2” indicate locations of the PCR marker loci scored on the independent sample of individual snails used to validate the Poolseq results (Table 1; PTC2 is same as locus 0 in Fig 1 of Blouin et al., 2022 [40]). “OPM-04” indicates the approximate location of RAPD marker identified by Knight et al. (1999 [20]), shown here for interest. (C) 10 kb sliding windows of average expected heterozygosity per segregating site across the same region as in panel B. *F*_st_ is correlated with heterozygosity, which probably varies owing to very low sequence similarity between haplotypes in this region (discussed further below).

The sliding window *F*_st_ analysis on the original Anderson-laboratory samples shows evidence for a region of association on LG16 that spans 7–8 Mb (Fig 1A). No other LGs contain peaks that are above the *F*_st_ ~0.06 cutoff. There appear to be two peaks within that LG16 region of association, with the right-hand peak (peak 1) including the ~450 kb region defined by Tennessen et al. (2020 [24]) as *PTC2* (Fig 1B). Aligning reads to all four PacBio assemblies produced essentially the same set of *F*_st_ peaks, with minor variations (S2 Fig). There is substantial variation in heterozygosity across this region that is correlated with *F*_st_ (Fig 1C).

### Validation of *F*_*st*_ peaks

**Fig 2:**
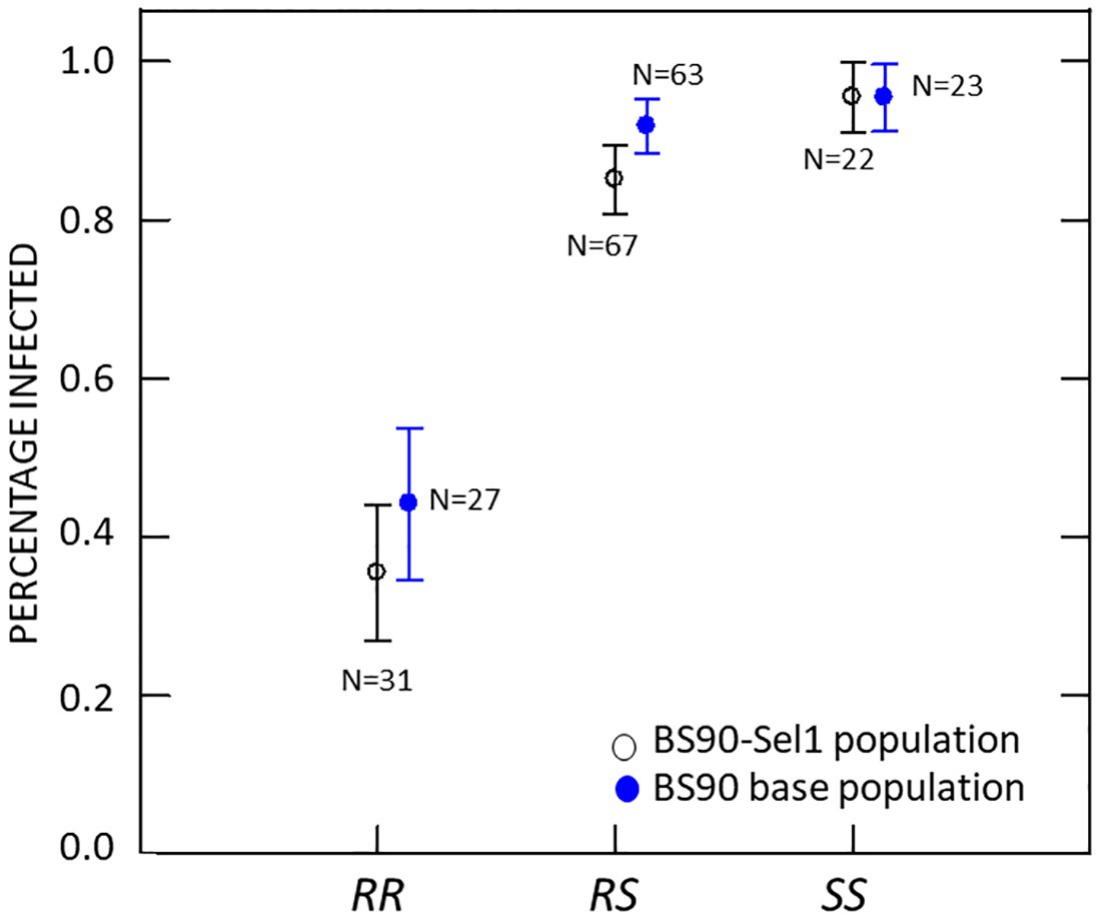
Percentage of snails of each genotype (*RR*, *RS* or *SS*) that became infected after challenge with 5–20 miracidia of SmLE. BS90 is the original population of BS90 snails. BS90-sel1 is a population of BS90 that were selected for resistance to SmLE for one generation. Error bars show the standard error of the proportion.

We validated the Poolseq results using PCR markers to type an independent sample of BS90 snails challenged by SmLE *S*. *mansoni*. The locations of the three PCR markers used are shown in Fig 1B. The PCR marker in the *PTC2* showed two, almost equally frequent alleles: a recessive allele (*R*) associated with resistance, and a dominant allele (*S*) associated with susceptibility (*R* allele frequency = 0.53 in BS90 and 0.55 in BS90-Sel1). The association between *PTC2* genotype and phenotype in the validation samples was strong and significant in both snail populations (Fig 2 and Table 1). Under a model of complete dominance by the *S* allele, and including both populations, odds of infection are 14.4 times higher for *SS* or *RS* individuals than for *RR* individuals (binary logistic regression P < 0.0005; odds ratios equal 12.9 and 16.7 for BS90-Sel and BS90 analyzed separately). In this analysis the population effect was not significant (P = 0.179), although the trend was in the direction expected from previous studies in our lab (S1 Data), at least within *RR* and *RS* genotypes (Fig 2).

**Table 1:** Odds ratios and statistical significance from logistic regressions of phenotype (infected or not) on genotype for each marker locus (*PTC2*, *Trough* or *Peak2*) in each population (each locus tested separately; see S1 Appendix for full output from all models and S2 Appendix for the raw data).

Population	*PTC2*	*Trough*	*Peak2*
BS90	16.7 (P < 0.0005)	2.9 (P = 0.016)	1.4 (P = 0.354)
BS90-Sel1	12.9 (P < 0.0005)	3.6 (P < 0.0005)	2.4 (P = 0.008)

In contrast to the strong effects at *PTC2*, we saw much weaker evidence for the causal locus occurring in peak 2, as the strength of association drops precipitously in going from *PTC2* to the *Trough* locus to the *Peak 2* locus (Table 1). The strength of association drops off more slowly with distance in BS90-Sel1 than in BS90. However, the BS90-Sel1 population shows higher linkage disequilibrium across this region than the BS90 base population, probably owing to a bottleneck during the selection experiment (S2 Table). A regression model that includes both *PTC2* and Peak 2 is only significant for *PTC2* (P < 0.0005), regardless of whether population is included as a cofactor (S1 Appendix). So, there is no evidence of a second causal locus at peak 2, and the association at peak 2 in the GWAS presumably results from linkage to *PTC2*.

So why are there two peaks in the sliding window *F*_st_ plot (Fig 1B) if we see no evidence for a causal locus in peak 2? The trough does not obviously result from an assembly error, for two reasons. Firstly, there was significant linkage disequilibrium among the three validation markers (*PTC2*, *Trough* and *Peak 2*) (S2 Table), so the three regions appear to be physically linked. Secondly, alignment of reads to all four PacBio assemblies shows the trough region in its putative location. However, the heights of the peaks in Fig 1B correlate positively with the heterozygosity in those regions, and the trough between the two peaks corresponds to a region of lower heterozygosity (Fig 1C). Therefore, we suspect the appearance of two peaks is an artifact. There is probably a broad region of association that spans peak 1 to peak 2, with the trough in *F*_st_ resulting from a lack of intermediate-frequency variants, which limits how high *F*_st_ can be.

### Phenotyping and sequencing of the inbred snail lines

When challenged with 10 miracidia of SmLE, the proportion of infected snails differs substantially among the three Blouin-laboratory inbred lines (same experimental conditions as used to challenge the outbred BS90 snails). Percentage infected ± standard error = 24% ± 5% (N = 71) for FRS11(RR),11% ± 4% (N = 66) for F6RR(RR), and 98% ± 1% (N = 181) for FSS5(SS). So, the phenotypes of these three lines correspond to expectation based on their *PTC2* genotypes. We do not have Bu et al.’s [35] iBS90(SS) line in our laboratory, so we don’t know its phenotype vs. SmLE.

The iBS90(SS) and FSS5(SS) assemblies are already published [35,40], so for this study we added PacBio assemblies for F6RR(RR) and FRS11(RR). Our F6RR(RR) genome assembly consists of 4,310 contigs covering 741,938,119 total length, with a fragment N50 size of 317,990, largest fragment size of 3,011,836, and mean coverage of 54. Our FRS11(RR) genome assembly consists of 4,968 contigs covering 916,856,302 bp total length, with a fragment N50 size of 8,258,380, largest fragment size of 41,646,661, and mean coverage of 156. The FRS11(RR) assembly is less fragmented in the *PTC2* region than the FRR6(RR) assembly, likely due to the increased depth of coverage and newer PacBio machine. Both new assemblies are comparable in contiguity to the previously reported FSS5(SS) assembly with N50 of 2.3 Mb.

### Genomic variation in the region of association

**Fig 3:**
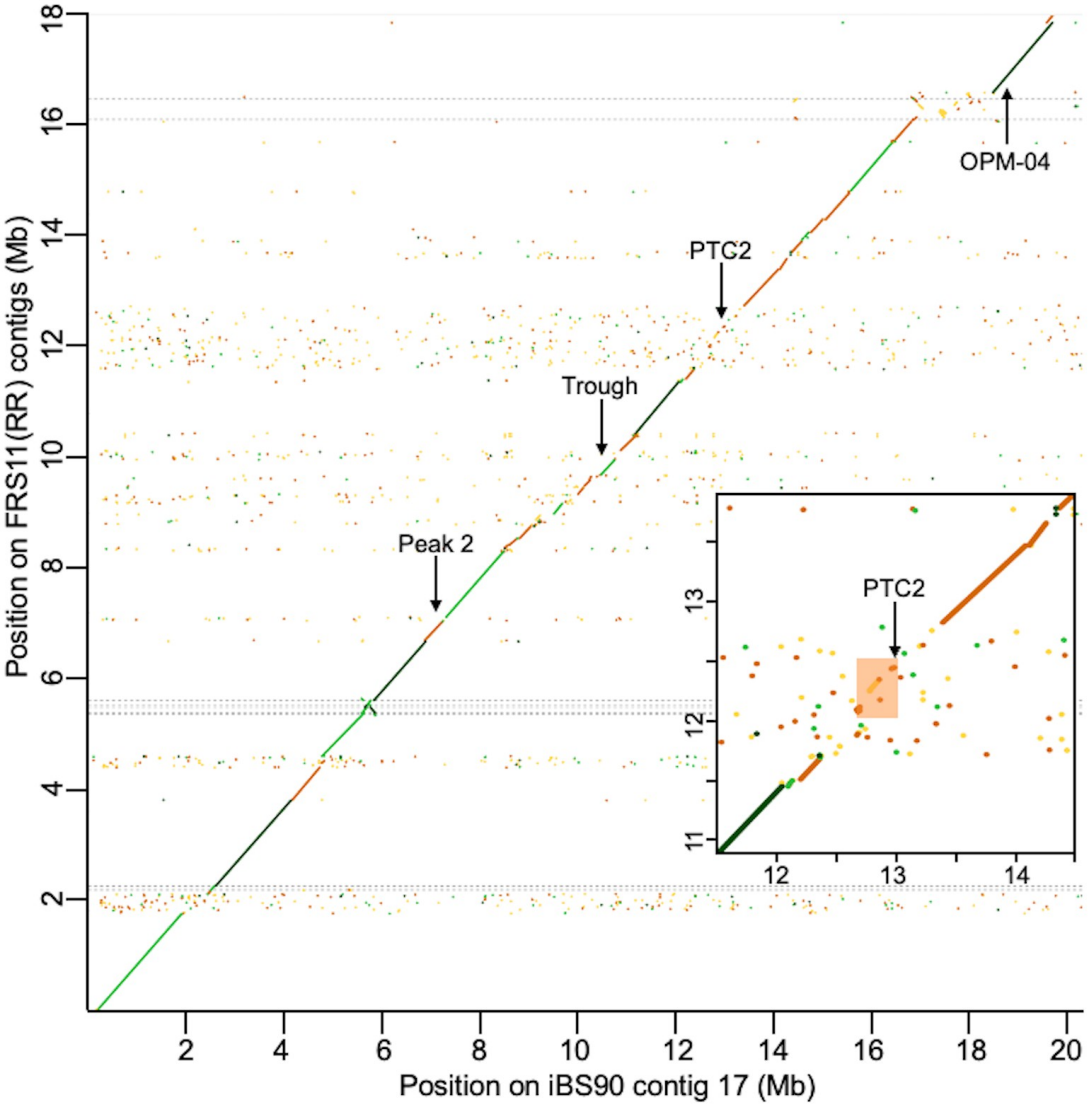
Dot plot comparing the 20 Mb length of iBS90(SS) contig 17 (which is *S* allele at *PTC2*) with the equivalent region in assembly FRS11(RR) (which is *R* allele at *PTC2*). Each dot represents 0.001% of the total width (200 bp). Sequence identity indicated by color: dark green = >75%, light green = 50–75%, brown = 25–50%, yellow = <25%. Dashed horizontal lines show contig boundaries in the FRS11(RR) assembly. Inset shows a closeup of 3 Mb surrounding the original *PTC2* region (orange box) described in [24]. Arrows indicate locations of our three PCR marker loci and OPM-04. Sequence identity between *RR* assemblies and *SS* assemblies is very low in the ~1 Mb block that contains the *PTC2* (S3 Fig). See S3 Fig for all six pairwise dot plots among the four assemblies. Plots were created in D-genies using default parameters [50].

From other studies, we know that nucleotide sequence identity among *B*. *glabrata PTC2* haplotypes from other snail populations is quite low, with large sections showing either no detectable homology or sequence divergence exceeding 50% between haplotypes [24,40]. We see a similar pattern between BS90 genome assemblies in this study (Figs 3 and S3). In our BS90 population, the *PTC2* is centered in an approximately 1 Mb block that shows very little sequence similarity between *R* and *S* haplotypes (Fig 3).

Interestingly, inbred lines FRS11(RR) and FSS5(SS) share very similar sequence starting from halfway through the trough to the beginning of the contig, which includes all of peak 2 (see S3 Fig, which shows all six pairwise dot plots among the four assemblies). FRS11(RR) and FSS5(SS) also share the same genotype at our Peak 2 marker locus. Given how different lines FSS5(SS) and FRS11(RR) are in phenotype, the fact that they appear to share the same sequence across peak 2, but different sequence across peak 1 (which includes the *PTC2*), adds additional evidence that the causal polymorphism is in peak 1, not in peak 2.

### Genes for single-pass transmembrane proteins may be involved

**Fig 4:**
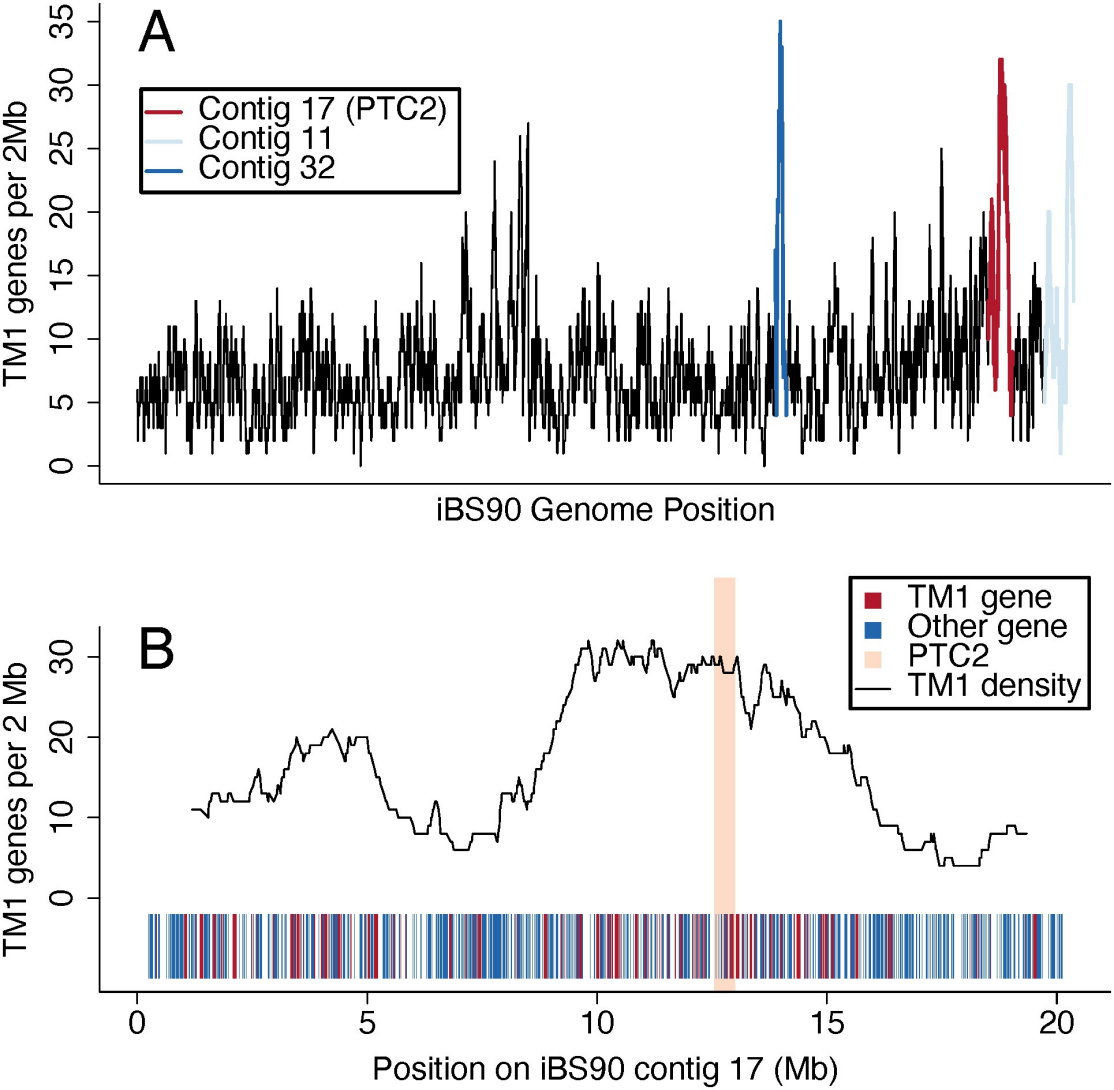
Density of single-pass transmembrane (TM1) genes in the iBS90 genome, as identified by TMHMM v. 2.0 [51], here measured in overlapping 2 Mb windows. (A) TM1 density along the entire genome. Along with Contigs 11 and 32, contig 17, which contains *PTC2*, has one of the highest densities of TM1 genes in the genome. (B) Density of TM1 genes along iBS90 contig 17 showing that the *PTC2* (orange box) sits within a wide region of high TM1 gene abundance, which roughly corresponds to peak 1 in the *F*_st_ plot of association with infection in Fig 1B.

**Fig 5:**
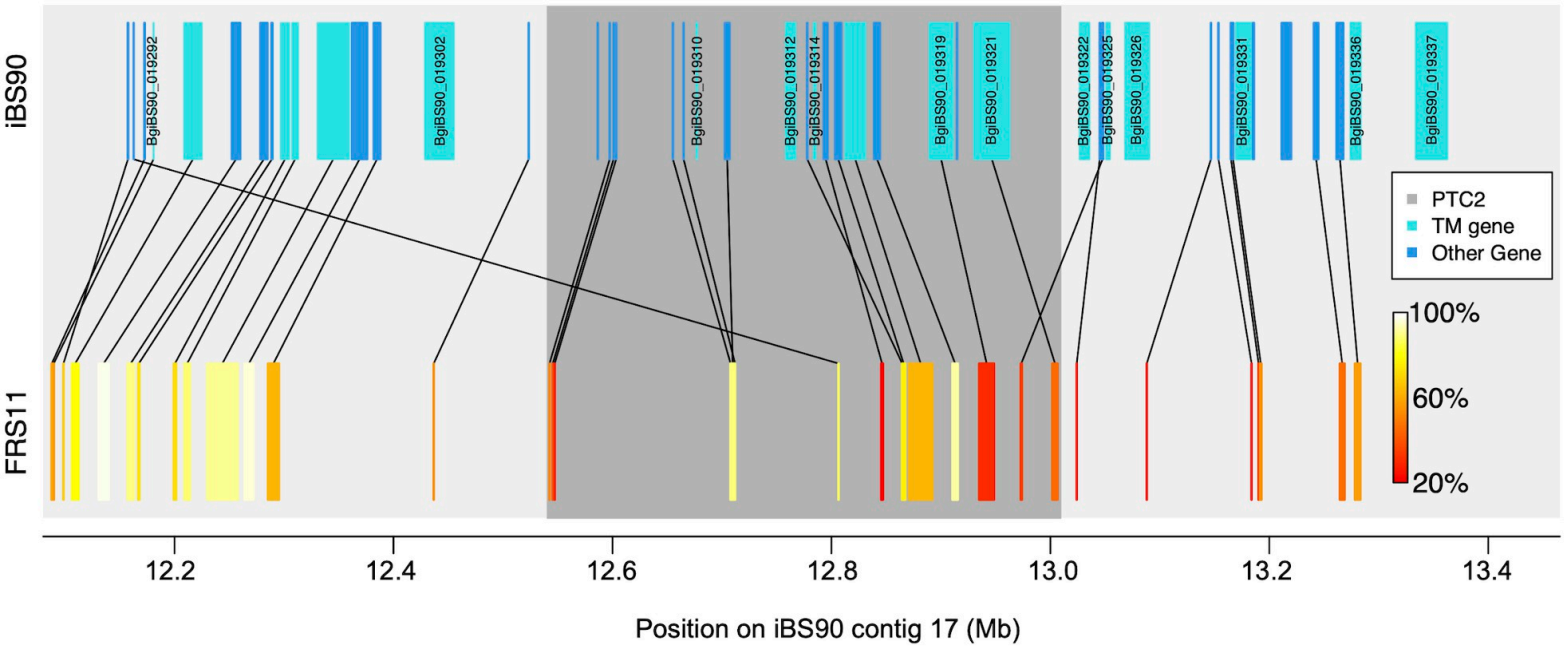
Gene orthology and synteny between the iBS90(SS) and FRS11(RR) assemblies in the 1.25 Mb region surrounding the *PTC2*. *PTC2* **=** approximately the region of low sequence similarity illustrated by the inset in Fig 3; *PTC2* = dark grey rectangle. For all annotated genes between 12.15 Mb and 13.4 Mb on Contig 17 from the iBS90(SS) assembly (blue rectangles), we used BLAST to identify putative orthologs in FRS11(RR) (heat-colored rectangles). Matches with at least 20% matching amino acid residues were considered putative orthologs, here color-coded by sequence similarity (% amino acid identity) and connected with black lines to show synteny. Several iBS90(SS) genes either have no ortholog or have a highly divergent ortholog (<60% similarity) on FRS11(RR). If these are also transmembrane genes (light blue) they are labeled. These represent particularly promising candidates to explain the observed dominant susceptibility. See S3 Table for additional information.

The part of LG16 that includes the *PTC2* (iBS90 contig 17) also shows an unusually high density of single-pass transmembrane (TM1) genes (Fig 4A), which may play a role in host-parasite interaction at the cell surface [22,24,25]. Notably, both the region of low sequence similarity and the cluster of single-pass transmembrane genes extend well beyond the boundaries of the original *PTC2* identified by [24], and encompass a wider region that roughly corresponds with peak 1 in the sliding window *F*_st_ plot (Fig 4B). Only 8% of genes in the *Biomphalaria* genome are TM1 genes, while 29% of the genes within ±2 Mb on either side of *PTC2* are TM1 (enrichment assessed with Fisher’s exact test: P = < 1e-15). In the ~1 Mb region where the *R* and *S* haplotypes share very low sequence similarity (see inset Fig 3), 35% of genes are TM1 (Fig 5; Fisher’s exact test P < 1e-06). Few genes in this region contain conserved protein domains or show clear homology to non-mollusc genes, presumably because they are rapidly evolving. They do, however, show homology to each other. Most of these genes fall into two large groups of paralogs, one represented by *PTC2* Gene 1 and the other represented by *PTC2* Gene 2 (S3 Table).

In this region, not only is there a high density of TM1 genes, but many are present on only one haplotype but not the other. Dominant susceptibility is consistent with a model in which the parasite can match a target on the *S* haplotype that is missing on the *R* haplotype (see section ‘Dominant Susceptibility’ in the Discussion below). Thus, genes that are present on the *S* haplotype but missing on the *R* haplotype would be compelling candidates to explain dominant susceptibility. Fig 5 identifies several such genes (present on the iBS90(SS) assembly and missing or highly divergent on the FRS11(RR) assembly).

## Discussion

### Allelic variation in the PTC2 strongly associates with resistance in BS90 snails

In our BS90 snail population we see a strong association between allelic variation at a marker in the *PTC2* region and resistance to infection by SmLE-population *S*. *mansoni* (odds of infection ~13–17 times greater for individuals that carry an *S* allele). The initial GWAS suggested an association in peak 2 several megabases away. But there is no evidence for a second locus affecting phenotype after accounting for *PTC2*, so we infer this to be merely a signal of linkage to *PTC2* (Table 1 and S1 Appendix). Furthermore, inbred lines FSS5(SS) and FRS11(RR) share the same peak-2 haplotype yet differ substantially in phenotype. So, rather than two peaks of association, we suspect there is just a broad region of association in the original GWAS snails, with peaks and troughs in *F*_st_ resulting from variation in heterozygosity, which limits how high *F*_st_ can be. This variation in heterozygosity could result in part from the low sequence identity across the region, as many reads might map only to one haplotype but not the other. For example, when we plot Illumina read depth on all four assemblies across the contig 17 region, we see substantial variation between assemblies in coverage over certain regions, including the trough (S4 Fig).

Why might the region of association around *PTC2* be so much wider in this study than the ~450 kb region we observed in Tennessen et al. [24]? In this study we used N = 96 snails per phenotype in the preliminary GWAS, whereas Tennessen et al. [24] used N = 600. So, a difference in power between the two studies could partly explain the broader region of association identified in this study. The width of linkage disequilibrium in the region of interest could also have been higher in the Anderson-laboratory samples used for the initial GWAS (which were raised in batches for another experiment), than in the Blouin-laboratory’s outbred populations.

Considering the results in [24,40], and this study, *PTC2* has emerged as a locus strongly associated with resistance in three independent experiments, each involving distinct host/parasite genotype combinations. While other *B*. *glabrata* loci are also associated with resistance in particular snail-schistosome combinations [22,23,35], *PTC2* consistently shows strong effects, suggesting broad importance of this genomic region to snail resistance (Table 2).

**Table 2:** Genomic regions identified in *B*. *glabrata* to date at which allelic variation explains variation in resistance to *S*. *mansoni*.

Locus	LG^a^	Snail population(s)	Parasite population	OR^b^	Reference
*PTC2*	16	Within 1316R1	SmPR1	15.9	Tennessen et al. 2020 [24]
		Between BS90 & M-line	SmPR1	4.8	Blouin et al 2022 [40]
		Within BS90	SmLE	14.4	This study
*OPM-04*	16	Between BS90 & M-line	SmPR1	NA^c^	Knight et al. 1999 [20]
				5.7^c^	Bu et al. 2022 [35]
				<1.3^c^	Blouin et al., 2022 [40]
*qRS-5*.*1*	5	Between BS90 & M-line	SmPR1	14.7	Bu et al. 2022 [35]
*qRS-2*.*1*	2	Between BS90 & M-line	SmPR1	4.4	Bu et al. 2022 [35]
*GRC/PTC1*	6	Within BgGUA	SmGUA	8.2	Tennessen et al. 2015 [22]
*sod1*	6	Within 1316R1	SmPR1	4.8	Tennessen et al. 2015 [23]
*RADres*	10	Within 1316R1	SmPR1	3.2	Tennessen et al. 2015 [23]

^a^Linkage group [35]

^b^Odds ratio between opposite homozygote genotypes (the square of the per-allele OR for additive models). Bu et al.’s [35] S3 Table reports OR for multiple SNP loci scored in each QTL region (qRS-5.1 and qRS-2.1), so we report the SNP having the largest value in each QTL.

^c^OR was not reported by Knight et al. [20] but implied to be indeterminately high as the resistant allele was never observed in infected snails. It was subsequently estimated using the putatively same snail populations and parasite population by Bu et al [35] and Blouin et al. [40].

### Transmembrane genes may be responsible for the association with resistance

In our four BS90 genome assemblies, the *PTC2* occurs in the middle of a ~1 Mb region that shows very low sequence identity between haplotypes bearing the *S* vs *R* allele (Fig 3). Furthermore, this block of divergent sequence is part of a much wider region that is structurally polymorphic and is highly enriched for single-pass transmembrane genes (Figs 4 and 5) (as was the original ~450 kb *PTC2* region identified by Tennessen et al. [24] in 1316R1-population snails challenged by SmPR1-population parasites). A cluster of seven TM1 genes on a different chromosome (the *GRC* region) showed strong association with resistance in Guadeloupean snails challenged by Guadeloupean *S*. *mansoni*, and RNAi knockdown of one of those genes (*grctm6*) made snails shed more cercariae [22,25]. Furthermore, genes in the *GRC* region appear to be involved in hemocyte recognition of pathogen-associated molecular patterns such as carbohydrates [26,27]. So, it seems likely that one or more TM1 genes in or near the *PTC2* are responsible for the association with resistance to infection observed here.

The larger region surrounding the *PTC2* is also very polymorphic among haplotypes from other *B*. *glabrata* populations (e.g. see Fig 6 in [40], which compares part of LG16 between our FSS5(SS) PacBio assembly and that of an M-line snail). This type of extreme polymorphism in both structure and sequence is typical of regions containing immune-relevant genes (e.g. [52]). So, it would not be surprising if there were multiple immune-relevant genes scattered across the larger region, even if the cluster of TM1 genes in the *PTC2* seem like obvious candidates in this particular dataset. In *Biomphalaria sudanica* from Africa, *PTC2* occurs within a region over 10 Mb in size that shows the highest nucleotide diversity in the genome as well as large structural rearrangements [53]. So, this putatively immune-relevant region has been maintained as highly diverse in structure and sequence for millions of years.

### Dominant susceptibility

The *PTC2* region was originally identified in the 1316R1 population of *B*. *glabrata* challenged by PR1-population *S*. *mansoni* [24]. In that population there were three very distinct haplotypes, which showed mostly additive effects among the six genotypes. In contrast, here we see two haplotypes and almost complete dominance of the *S* allele (Fig 2). Dominant susceptibility suggests an uncommon model of host-pathogen interaction, such as inverse gene-for-gene matching [54]. Here infection proceeds only if the parasite can “match” an allele in the host, and the host evades infection by having alternate or null (missing) versions of that receptor (e.g. as with the HIV-resistant null allele at the CCR5 receptor on human T-cells [55]). Therefore, it is possible that some molecule produced by invading schistosomes binds to the snail protein, and that this binding either suppresses the host immune response or serves to camouflage the parasite. Indeed, it has been repeatedly suggested that schistosomes use molecular mimicry and immunosuppression to evade snail immune systems [19,56]. Under a model of dominant susceptibility, genes in the vicinity of *PTC2* that are present on *S* haplotypes, but absent or highly divergent on *R* haplotypes, would be particularly interesting candidates. Fig 5 shows that multiple TM1 genes in the ~1 Mb region around *PTC2* fit this pattern.

### Relevance to other studies on BS90 snails

Blouin et al. [40] observed heterozygote excess at *PTC2* in multiple, independent BS90 by M-line intercross populations, which raises the possibility of some kind of balancing selection at the locus. Thus, it is interesting that we observed a slight, albeit non-significant heterozygote excess at *PTC2* in both populations in this study (*F*_IS_ = -0.12 and -0.15 in BS90-Sel1 and BS90, respectively). The very even allele frequencies (frequency of *R* allele = 0.53–0.55) would also be consistent with balancing selection at this locus.

In this study, we examined variation *within* the BS90 population in resistance to the SmLE population of *S*. *mansoni*. BS90 is 100% resistant to several other populations of *S*. *mansoni*, including SmPR1. Other snail populations such as M-line are very susceptible to SmPR1. Knight et al. [20] concluded that the difference between BS90 and M-line snails in resistance to SmPR1 parasites segregated as a single locus trait with the BS90 allele dominant. Furthermore, in a QTL-mapping cross they observed a RAPD marker (OPM-04) that segregated with the resistance phenotype. Marker OPM-04 maps to within ~5–6 Mb of the *PTC2* (Fig 2), which suggested the hypothesis that genes in the *PTC2* are involved. We recently used marker-assisted backcrossing to drive the BS90 *PTC2* region into an M-line genetic background, proving that the *PTC2* does *not* explain the difference between M-line and BS90 in resistance to SmPR1 [40]. We instead observed a significant effect in the opposite direction. Furthermore, neither we nor Bu et al. [35] could replicate the association with the OPM-04 region in F2 QTL crosses between BS90 and M-line (though in Bu et al.’s S3 Table, they report an additional variant within the 1.2 kb OPM-04 locus that was not part of their QTL analysis, but that does appear to show a small effect; reported in Table 2 above). Bu et al. used iBS90(SS) in their mapping cross, and we used FSS5(SS), both of which are *SS* at *PTC2*. It is possible that Knight et al. [20] used an *RR* line of BS90 in their experiments, which might explain the highly-penetrant, dominant phenotype they observed in their F2 snails. Alternately, a second etiological locus near OPM-04, rather than its linkage to *PTC2*, could in some crosses explain this marker’s association with variance between BS90 and M-line in resistance to PR1-population *S*. *mansoni*.

Although BS90 snails are treated in the literature as if they are a uniform ‘strain’, it is worth noting that BS90 actually appears to be a genetically variable, outbred population (also see evidence of this in [10]). Given this ‘strain’ of snails has been passed among laboratories for decades, researchers should keep in mind that one lab’s “BS90” may not be identical to another’s. This variation could explain inconsistent results among laboratories, such as Blouin et al.’s [40] and Bu et al.’s [35] differing ability to replicate Knight et al.’s [20] result. Thus, we encourage researchers to include information on the origin and history of the BS90 population they used (e.g. source and date obtained) in any publications using this named population of snails.

### TM1 genes and genotype-by-genotype models of snail-parasite interaction

Resistance of *B*. *glabrata* snails to *S*. *mansoni* is highly heritable and easily selected for [7,9,22,57]. Similarly, lab populations of *S*. *mansoni* can be selected for either higher or lower infectivity to particular lines of snails [7,58]. Snail populations that are highly resistant to one *S*. *mansoni* population often remain highly susceptible to other *S*. *mansoni* populations, and *vice versa* for parasite populations’ abilities to infect different snail populations [7,9,10,59].

In Théron *et al*. [36] we showed that a simple system of genotype-matching could explain the shapes of dose-response curves (percentage of snails infected vs dose of parasite exposure). Also, taking both snails and schistosomes from the wild in Guadeloupe into the lab created a highly resistant sub-population of snails, which we hypothesize resulted from loss of a matching allele in the parasite population [36]. Mapping this variation revealed the *GRC* region and its TM1 genes [22]. Subsequent studies have repeatedly pointed to the *PTC2*, another TM1-gene-enriched region having highly-penetrant loci that control snail susceptibility/resistance (Table 2). On the schistosome side, QTL mapping in F2 crosses between populations of *S*. *mansoni* that differ in their ability to infect BS90 snails revealed a single genomic region of very strong effect [60]. Therefore, the high heritability of susceptibility in snails and of infectiousness in schistosomes, the evidence for large-effect QTLs on both sides, and the ubiquitous snail-population by Schistosome-population interaction in compatibility, all suggest that compatibility polymorphisms are driven by some type of matching-alleles or gene-for-gene interactions (e.g. [61,62]). We hypothesize that molecular matching between highly diverse TM1 genes on the snail side, and some still-unknown molecules on the schistosome side, are behind these patterns.

### Relevance to public health

We have identified TM1 loci as likely candidates on the snail side to explain the ubiquitous genotype-by-genotype interactions in compatibility we see between snail and schistosome populations. We currently have little idea which molecules are important on the schistosome side, but identifying the schistosome products that interact with snail TM1 proteins could reveal key mechanisms by which schistosomes overcome host defenses. This information could be used to genetically modify snails to make them less able to transmit the parasite in the field. Note that rendering snails completely resistant to infection is not essential for control—in an integrated approach involving mass drug administration, even a partial reduction in transmission rate at the snail stage could have large epidemiological effects [63–65]. So, the translational effects of finally identifying the genes behind these snail-schistosome compatibility polymorphisms could be significant.

### Summary

A preliminary GWAS on BS90 snails using hybrid *S*. *mansoni* (F2s from a cross between SmLE and SmBRE) showed a broad peak of association on LG16. This peak encompasses the smaller *PTC2* region previously identified by Tennessen et al. (2020) [24]. Independent validation using BS90 challenged by pure SmLE showed a strong signal of association at a marker locus in the *PTC2*. At this locus, the allele that increases susceptibility (*S*) is almost completely dominant to the allele that reduces susceptibility (*R*). This pattern suggests a model in which the parasite must match some molecule in the host to successfully infect. Haplotypes bearing the *S* and *R* alleles share very little sequence identity in the ~1 Mb region immediately surrounding the *PTC2*. Furthermore, that region and the broader region that stretches for several Mb in either direction is not only highly variable in structure and sequence but is highly enriched for single-pass transmembrane genes. Therefore, we speculate that dominant susceptibility results from the presence of one or more TM1 genes on the *S*-bearing haplotype that are absent or very different on the *R*-bearing haplotype. If so, then identifying the genes involved might give insight into mechanisms used by the parasite to overcome host defenses, which could suggest new ways to break the cycle of transmission of schistosomiasis.

## Supporting information

S1 Fig Significance of *F*_st_ peak height. (DOCX)

S2 Fig Sliding window *F*_st_ and heterozygosity with reads aligned to the three other PacBio assemblies. (DOCX)

S3 Fig Pairwise dot plots among the four PacBio assemblies for the iBS90 contig 17 region. (JPG)

S4 Fig Mean read depth across iBS90 contig 17 region for reads aligned to all four PacBio assemblies. (DOCX)

S1 Table Primer and amplicon information for the three marker loci. (XLSX)

S2 Table Pairwise linkage disequilibrium estimates among the three marker loci. (DOCX)

S3 Table Descriptions of the genes identified in Fig 5. (XLSX)

S1 Appendix Output from logistic regressions. (DOCX)

S2 Appendix Raw data used in logistic regressions. (XLSX)

S1 Data Data showing the BS90-Sel1 population is more resistant than the BS90 base population. (XLSX)
