# Supplementary material for: Susceptibility of BS90 Biomphalaria glabrata snails to infection by SmLE Schistosoma mansoni segregates as a dominant allele in a cluster of polymorphic genes for single-pass transmembrane proteins
Source: PLoS Negl Trop Dis. 2024 Sep 16;18(9):e0012474. doi: 10.1371/journal.pntd.0012474 (PMC11426442; doi:10.1371/journal.pntd.0012474)

**S1 Figure (2 parts: A and B)**

**Part A.** *Library pooling procedure for the main GWAS and for creating an empirical F*_st_ *cutoff for significance of peak height.*

The snails for the GWAS were obtained from an experiment in the Anderson laboratory in which snails were challenged by F2 miracidia from a cross between SmLE and SmBRE population schistosomes. We used snails from four batches, labelled in the figure below as F2a1, F2a2, and so on. *a* and *b* refer to snails from two independent experiments (reciprocal crosses between SmLE and SmBRE), while 1 and 2 refer to groups of snails challenged on different days in each experiment. We extracted DNA from 24 infected snails (labelled S) and 24 uninfected snails (labelled R) from each batch. We divided these groups of 24 into two subpools of 12 snails each, and then created Illumina libraries from each subpool (labelled p1 to p16). For the GWAS in the main manuscript we combined the reads from the eight R libraries to create an uninfected pool (i.e. subpools p1,p2,p5,p6,p9,p10,p13 and p14), and combined the reads from the eight S libraries to create an infected pool (i.e. subpools p3,p4, p7,p8, p11,p12,p15 and p16). We then did sliding window *F_st_* analysis between these two pools. For significance testing we pooled reads from the eight even-numbered libraries into one group (p2,p4,p6 etc..), and reads from the eight odd-numbered libraries in another group (p1,p3,p5,etc…). This created two groups, each of which was a 50:50 mix of infected and uninfected snails. We then estimated the maximum height of *F_st_* peaks that arose when these two groups were compared (see Part B below).


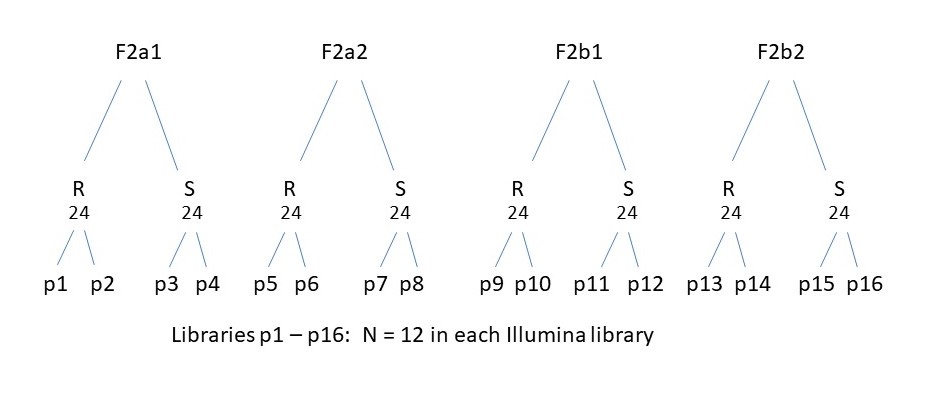


**Part B.**  *Empirical F_st_ cutoff for significance.*

Sliding window *F_st_* analysis between two pools that each contain a mixture of 4 infected subpools and 4 non-infected subpools (*F_st_* in 10 kb windows, sliding 5 kb each time, as in main manuscript Figure 1A). Panels A-D represent alignment of the same reads to Pacbio assemblies iBS90(SS), FSS5(SS), FRS11(RR) and FRR6(RR), respectively. From these plots we conclude that only peaks substantially higher than *F_st_* ~ 0.06 are likely to represent allele frequency differences between pools that are actually associated with infection status.


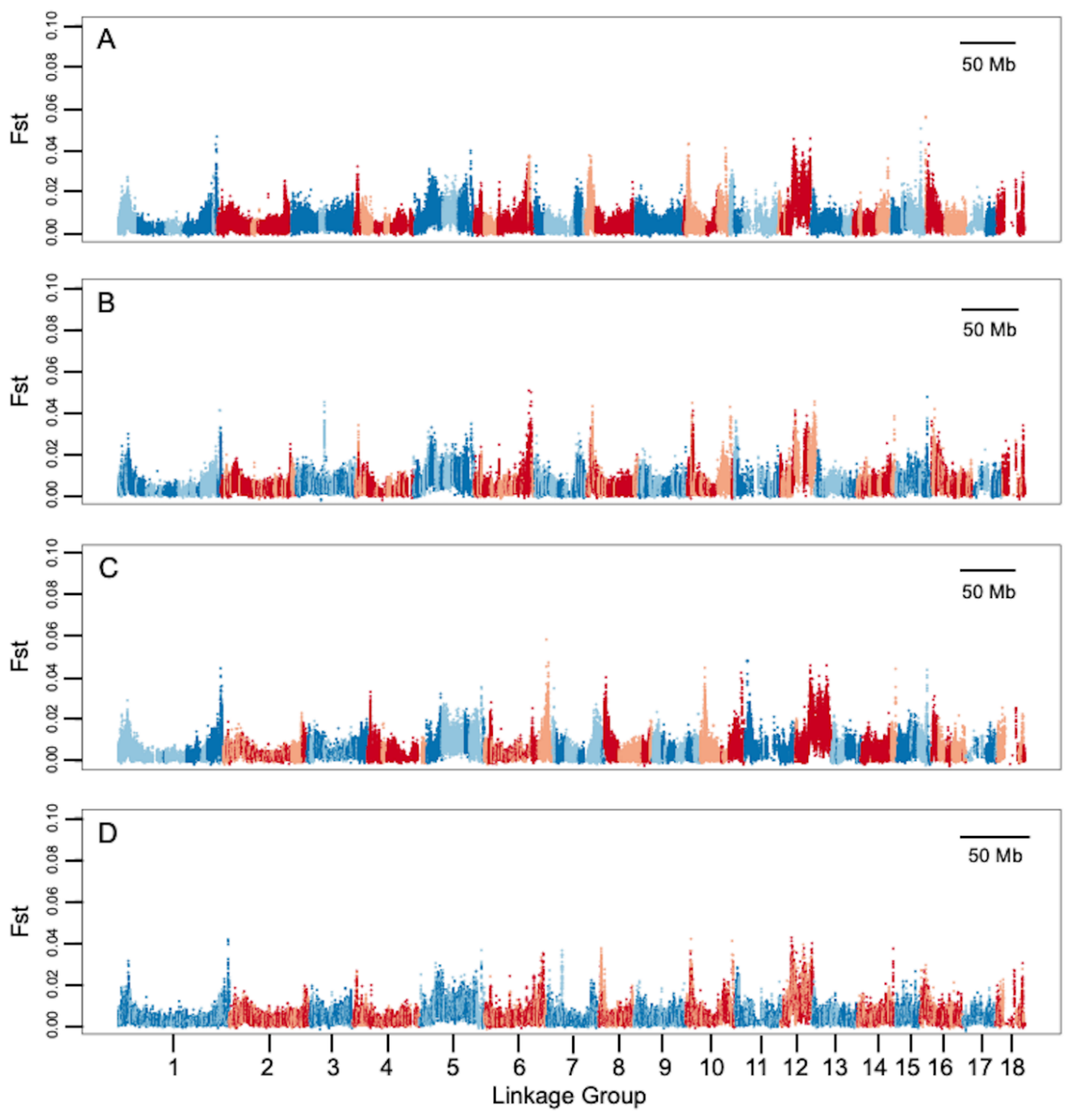

Supplement: S1 Fig — (DOCX) [file pntd.0012474.s001.docx]
