# Supplementary material for: Susceptibility of BS90 Biomphalaria glabrata snails to infection by SmLE Schistosoma mansoni segregates as a dominant allele in a cluster of polymorphic genes for single-pass transmembrane proteins
Source: PLoS Negl Trop Dis. 2024 Sep 16;18(9):e0012474. doi: 10.1371/journal.pntd.0012474 (PMC11426442; doi:10.1371/journal.pntd.0012474)

**S2 Fig.** Sliding window *F*_st_ over whole genome (panel A of each assembly), *F*_st_ over the region corresponding to iBS90(SS) contig 17 (panel B for each assembly) and heterozygosity over the contig 17 region (panel C for each assembly), for reads aligned to each of the additional genomes (FRS11(RR), F6RR(RR) and FSS5(SS) – identified by label on the X axis of each figure) (see main manuscript Fig 1 for reads aligned to iBS90(SS)). Alternating orange and red dots indicate contig boundaries in each assembly. The general pattern of *F*_st_ and heterozygosity across the region is the same for reads aligned to all four genomes.


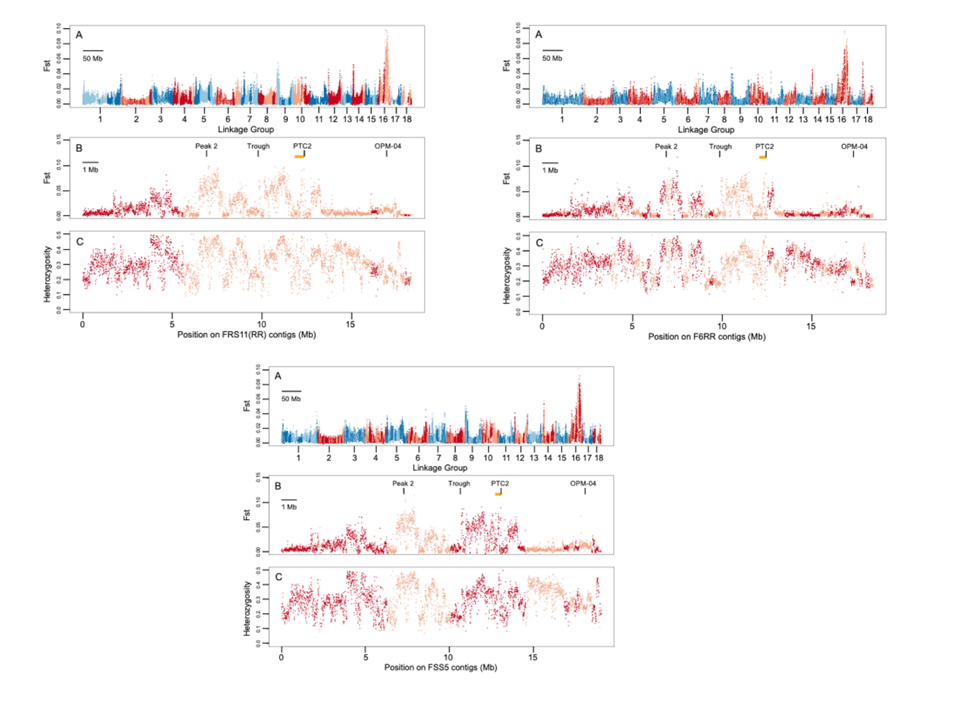

Supplement: S2 Fig — (DOCX) [file pntd.0012474.s002.docx]
