# Supplementary figures and images for: Susceptibility of BS90 Biomphalaria glabrata snails to infection by SmLE Schistosoma mansoni segregates as a dominant allele in a cluster of polymorphic genes for single-pass transmembrane proteins
Source: PLoS Negl Trop Dis. 2024 Sep 16;18(9):e0012474. doi: 10.1371/journal.pntd.0012474 (PMC11426442; doi:10.1371/journal.pntd.0012474)

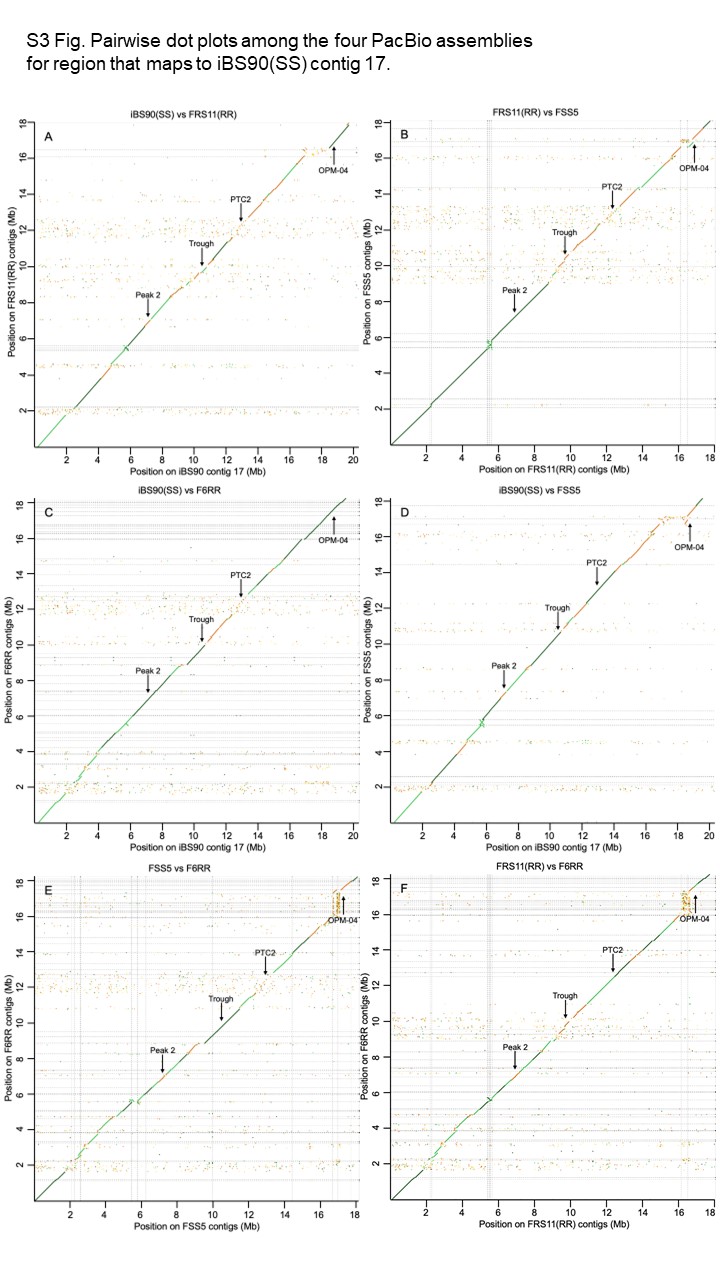

Supplement: S3 Fig — (JPG) [file pntd.0012474.s003.jpg]
