# Supplementary material for: Susceptibility of BS90 Biomphalaria glabrata snails to infection by SmLE Schistosoma mansoni segregates as a dominant allele in a cluster of polymorphic genes for single-pass transmembrane proteins
Source: PLoS Negl Trop Dis. 2024 Sep 16;18(9):e0012474. doi: 10.1371/journal.pntd.0012474 (PMC11426442; doi:10.1371/journal.pntd.0012474)

**S4 Figure**. Mean read depth in 10 Kb windows across iBS90 contig 17. The top two panels are from Figure 1 in the main manuscript, and show variation in *F*_st_ and expected heterozygosity across iBS90 contig 17. The four lower panels show read depth for the Illumina reads (all 16 libraries) aligned to each of the four PacBio assemblies. Notice that read depth in particular sections is variable among assemblies. For example, when reads are mapped to FRS11(RR), there is a drop in coverage across the region of low *F*_st_ between peaks 1 and 2. We suspect that low sequence identity between haplotypes across this region results in many reads mapping to some haplotypes but not others, which could reduce the number of intermediate frequency haplotypes that can be called by the variant-calling software, in turn causing variation in how high *F*_st_ can be.


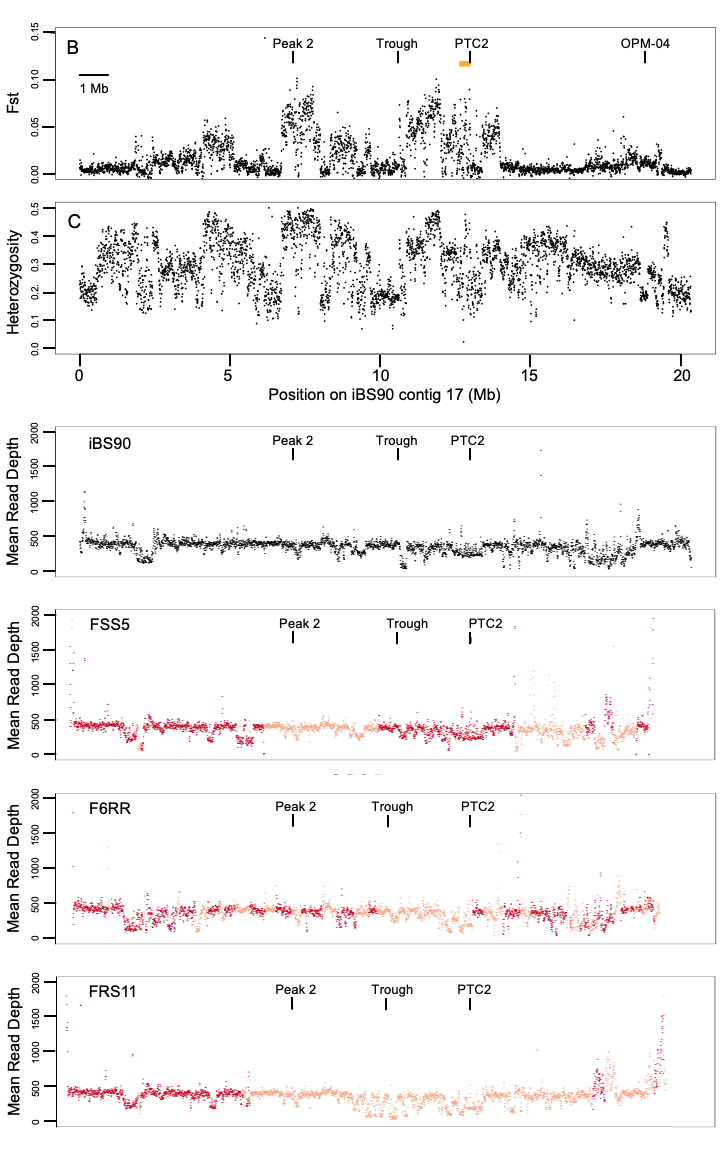

Supplement: S4 Fig — (DOCX) [file pntd.0012474.s004.docx]
