# Supplementary material for: Susceptibility of BS90 Biomphalaria glabrata snails to infection by SmLE Schistosoma mansoni segregates as a dominant allele in a cluster of polymorphic genes for single-pass transmembrane proteins
Source: PLoS Negl Trop Dis. 2024 Sep 16;18(9):e0012474. doi: 10.1371/journal.pntd.0012474 (PMC11426442; doi:10.1371/journal.pntd.0012474)
Supplement: S2 Table — (DOCX) [file pntd.0012474.s006.docx]

**S2 Table.** Pairwise linkage disequilibrium (LD) estimates among the three loci (maximum likelihood estimates assuming Hardy-Weinberg Equilibirum; estimated using GenAlEx 6.5). Notice that the strength of LD drops off more quickly with distance in the BS90 population than in the BS90-Sel1 population (presumably because of a bottleneck during the one round of selection for resistance to SmLE in BS90-Sel1).

| **Locus Pair** | **Population** | **D^a^** | **r^b^** | **ChiSq D^c^** | **P** | **Significance^d^** |
| --- | --- | --- | --- | --- | --- | --- |
| ptc2 / trough | BS90-Sel1 | 0.104 | 0.464 | 30.782 | 0.000 | *** |
| ptc2 / peak2 | BS90-Sel1 | 0.088 | 0.385 | 18.631 | 0.000 | *** |
| trough / peak2 | BS0-Sel1 | 0.082 | 0.394 | 19.971 | 0.000 | *** |
|  |  |  |  |  |  |  |
| ptc2 / trough | BS90 | 0.137 | 0.596 | 43.057 | 0.000 | *** |
| ptc2 / peak2 | BS90 | 0.014 | 0.064 | 0.432 | 0.511 | ns |
| trough / peak2 | BS90 | 0.055 | 0.262 | 5.757 | 0.016 | * |

^a^Standardized coefficient of Linkage Disequilibrium

^b^Correlation between alleles

^c^Chi-square test for D with 1 d.f.

^d^*** = P < 0.0001, * = P < 0.05
