# Supplementary material for: Susceptibility of BS90 Biomphalaria glabrata snails to infection by SmLE Schistosoma mansoni segregates as a dominant allele in a cluster of polymorphic genes for single-pass transmembrane proteins
Source: PLoS Negl Trop Dis. 2024 Sep 16;18(9):e0012474. doi: 10.1371/journal.pntd.0012474 (PMC11426442; doi:10.1371/journal.pntd.0012474)
Supplement: S1 Appendix — (DOCX) [file pntd.0012474.s008.docx]

**S1 Appendix.** Output from logistic regressions of infection status on genotype at the three marker loci. See S2 Appendix for the raw data used in these analyses.

**Section 1.** Graphical illustration of the genotype effects tested below (Blue circles = BS90, Black circles = BS90-Sel 1). At each locus the allele that appears to confer the lowest susceptibility is labelled *R*. Panel on the left is the same as figure 2 in the main manuscript.


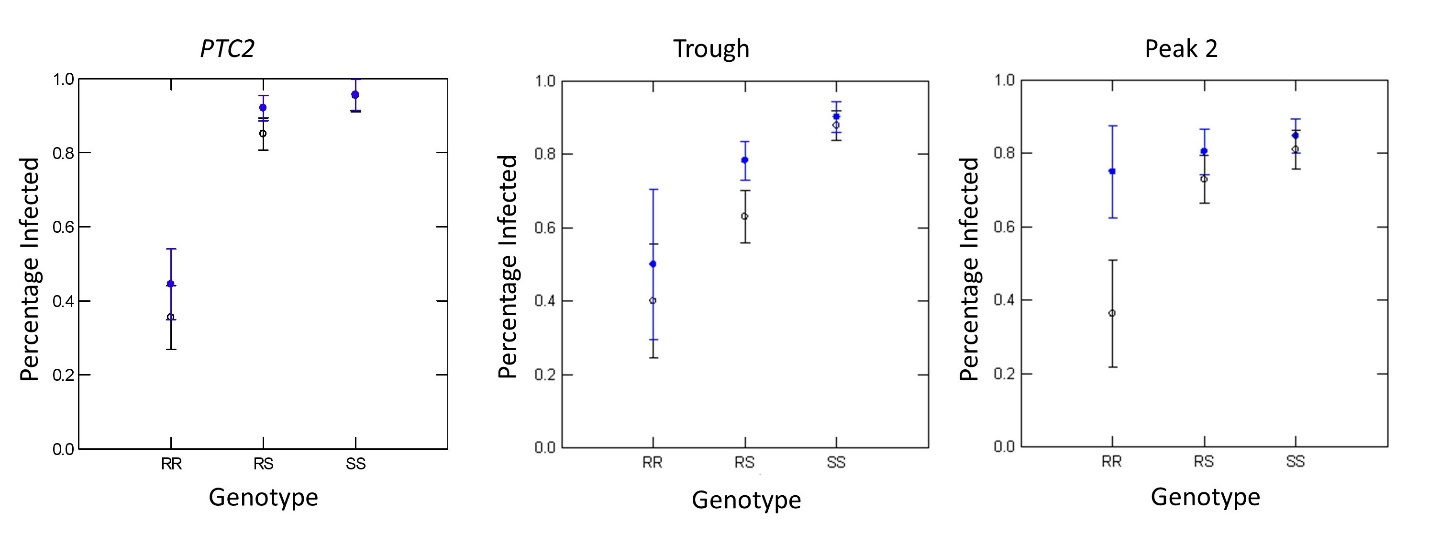


**Section 2:** Logistic regressions for each locus separately (*PTC2*, Trough and Peak 2) or for PTC2 and Peak 2 together. Analyses are done with both populations in the model (to test the population effect) or for each population separately. For each locus we show output from the model that gave the best fit (dominance for PTC2, additive for Trough and Peak 2).

**Section 2a**. Each locus separately, both populations in the model.

***PTC2*, both populations, dominance model** (i.e. Genotype = *RR* vs *S_*; Population = BS90 vs BS90-Sel1). See also Figure 2 in the main manuscript.

| **Parameter Estimates** | | | | | | |
| --- | --- | --- | --- | --- | --- | --- |
| **Parameter** | **Estimate** | **Standard Error** | **Z** | **p-Value** | **95% Confidence Interval** | |
|  |  |  |  |  | **Lower** | **Upper** |
| Constant | -2.006 | 0.295 | -6.795 | 0.000 | -2.585 | -1.428 |
| PTC2 Genotype | 2.668 | 0.374 | 7.129 | 0.000 | 1.935 | 3.402 |
| Population | -0.506 | 0.377 | -1.344 | 0.179 | -1.244 | 0.232 |

| **Odds Ratio Estimates** | | | | |
| --- | --- | --- | --- | --- |
| **Parameter** | **Odds Ratio** | **Standard Error** | **95% Confidence Interval** | |
|  |  |  | **Lower** | **Upper** |
| PTC2 Genotype | 14.414 | 5.395 | 6.921 | 30.017 |
| Population | 0.603 | 0.227 | 0.288 | 1.261 |

***Trough*, both populations, additive model** (i.e. genotype = dose of *R* alleles, 0,1 or 2)

| **Parameter Estimates** | | | | | | |
| --- | --- | --- | --- | --- | --- | --- |
| **Parameter** | **Estimate** | **Standard Error** | **Z** | **p-Value** | **95% Confidence Interval** | |
|  |  |  |  |  | **Lower** | **Upper** |
| Constant | -1.833 | 0.296 | -6.184 | 0.000 | -2.414 | -1.252 |
| Population | -0.536 | 0.336 | -1.592 | 0.111 | -1.195 | 0.124 |
| Trough Genotype | 1.194 | 0.270 | 4.420 | 0.000 | 0.664 | 1.723 |

| **Odds Ratio Estimates** | | | | |
| --- | --- | --- | --- | --- |
| **Parameter** | **Odds Ratio** | **Standard Error** | **95% Confidence Interval** | |
|  |  |  | **Lower** | **Upper** |
| Population | 0.585 | 0.197 | 0.303 | 1.132 |
| Trough Genotype | 3.300 | 0.891 | 1.944 | 5.603 |

***Peak 2*, both populations, additive model**

| **Parameter Estimates** | | | | | | |
| --- | --- | --- | --- | --- | --- | --- |
| **Parameter** | **Estimate** | **Standard Error** | **Z** | **p-Value** | **95% Confidence Interval** | |
|  |  |  |  |  | **Lower** | **Upper** |
| Constant | -1.415 | 0.271 | -5.216 | 0.000 | -1.947 | -0.883 |
| Population | -0.478 | 0.330 | -1.447 | 0.148 | -1.125 | 0.169 |
| Peak2 genotype | 0.621 | 0.236 | 2.632 | 0.008 | 0.159 | 1.084 |

| **Odds Ratio Estimates** | | | | |
| --- | --- | --- | --- | --- |
| **Parameter** | **Odds Ratio** | **Standard Error** | **95% Confidence Interval** | |
|  |  |  | **Lower** | **Upper** |
| Population | 0.620 | 0.205 | 0.325 | 1.184 |
| Peak2 genotype | 1.861 | 0.440 | 1.172 | 2.957 |

**Section 2b**. PTC2, Peak 2 and both populations in the same model.

| **Parameter Estimates** | | | | | | |
| --- | --- | --- | --- | --- | --- | --- |
| **Parameter** | **Estimate** | **Standard Error** | **Z** | **p-Value** | **95% Confidence Interval** | |
|  |  |  |  |  | **Lower** | **Upper** |
| Constant | -2.158 | 0.354 | -6.098 | 0.000 | -2.851 | -1.464 |
| Population | -0.547 | 0.386 | -1.418 | 0.156 | -1.304 | 0.209 |
| Peak2 Genotype | 0.312 | 0.285 | 1.095 | 0.274 | -0.247 | 0.872 |
| PTC2 Genotype | 2.596 | 0.386 | 6.725 | 0.000 | 1.840 | 3.353 |

| **Odds Ratio Estimates** | | | | |
| --- | --- | --- | --- | --- |
| **Parameter** | **Odds Ratio** | **Standard Error** | **95% Confidence Interval** | |
|  |  |  | **Lower** | **Upper** |
| Population | 0.578 | 0.223 | 0.271 | 1.233 |
| Peak2 Genotype | 1.367 | 0.390 | 0.781 | 2.392 |
| PTC2 Genotype | 13.417 | 5.180 | 6.295 | 28.593 |

**Section 2c.** Each locus separately for each population (these data went into main manuscript Table 1.)

***PTC2*, BS90 only (dominance model)**

| **Parameter Estimates** | | | | | | |
| --- | --- | --- | --- | --- | --- | --- |
| **Parameter** | **Estimate** | **Standard Error** | **Z** | **p-Value** | **95% Confidence Interval** | |
|  |  |  |  |  | **Lower** | **Upper** |
| Constant | -2.590 | 0.423 | -6.120 | 0.000 | -3.420 | -1.761 |
| PTC2 Genotype | 2.813 | 0.574 | 4.904 | 0.000 | 1.689 | 3.938 |

| **Odds Ratio Estimates** | | | | |
| --- | --- | --- | --- | --- |
| **Parameter** | **Odds Ratio** | **Standard Error** | **95% Confidence Interval** | |
|  |  |  | **Lower** | **Upper** |
| PTC2 Genotype | 16.667 | 9.562 | 5.414 | 51.311 |

***PTC2*, BS90-Sel1 only (dominance model)**

| **Parameter Estimates** | | | | | | |
| --- | --- | --- | --- | --- | --- | --- |
| **Parameter** | **Estimate** | **Standard Error** | **Z** | **p-Value** | **95% Confidence Interval** | |
|  |  |  |  |  | **Lower** | **Upper** |
| Constant | -1.959 | 0.322 | -6.082 | 0.000 | -2.590 | -1.328 |
| PTC2 Genotype | 2.557 | 0.495 | 5.169 | 0.000 | 1.587 | 3.526 |

| **Odds Ratio Estimates** | | | | |
| --- | --- | --- | --- | --- |
| **Parameter** | **Odds Ratio** | **Standard Error** | **95% Confidence Interval** | |
|  |  |  | **Lower** | **Upper** |
| PTC2 Genotype | 12.893 | 6.377 | 4.890 | 33.990 |

**Trough, BS90 only (additive model)**

| **Parameter Estimates** | | | | | | |
| --- | --- | --- | --- | --- | --- | --- |
| **Parameter** | **Estimate** | **Standard Error** | **Z** | **p-Value** | **95% Confidence Interval** | |
|  |  |  |  |  | **Lower** | **Upper** |
| Constant | -2.248 | 0.436 | -5.158 | 0.000 | -3.102 | -1.394 |
| Trough Gentoype | 1.050 | 0.434 | 2.420 | 0.016 | 0.200 | 1.900 |

| **Odds Ratio Estimates** | | | | |
| --- | --- | --- | --- | --- |
| **Parameter** | **Odds Ratio** | **Standard Error** | **95% Confidence Interval** | |
|  |  |  | **Lower** | **Upper** |
| Trough Genotype | 2.856 | 1.239 | 1.221 | 6.683 |

**Trough, BS90-Sel1 only (additive model)**

| **Parameter Estimates** | | | | | | |
| --- | --- | --- | --- | --- | --- | --- |
| **Parameter** | **Estimate** | **Standard Error** | **Z** | **p-Value** | **95% Confidence Interval** | |
|  |  |  |  |  | **Lower** | **Upper** |
| Constant | -1.897 | 0.339 | -5.590 | 0.000 | -2.563 | -1.232 |
| Trough Genotype | 1.282 | 0.346 | 3.704 | 0.000 | 0.604 | 1.960 |

| **Odds Ratio Estimates** | | | | |
| --- | --- | --- | --- | --- |
| **Parameter** | **Odds Ratio** | **Standard Error** | **95% Confidence Interval** | |
|  |  |  | **Lower** | **Upper** |
| Trough Genotype | 3.603 | 1.247 | 1.829 | 7.100 |

**Peak 2, BS90 only (additive model)**

| **Parameter Estimates** | | | | | | |
| --- | --- | --- | --- | --- | --- | --- |
| **Parameter** | **Estimate** | **Standard Error** | **Z** | **p-Value** | **95% Confidence Interval** | |
|  |  |  |  |  | **Lower** | **Upper** |
| Constant | -1.687 | 0.344 | -4.900 | 0.000 | -2.362 | -1.012 |
| Peak2 genotype | 0.331 | 0.357 | 0.927 | 0.354 | -0.368 | 1.030 |

| **Odds Ratio Estimates** | | | | |
| --- | --- | --- | --- | --- |
| **Parameter** | **Odds Ratio** | **Standard Error** | **95% Confidence Interval** | |
|  |  |  | **Lower** | **Upper** |
| Peak2 genotype | 1.392 | 0.496 | 0.692 | 2.800 |

**Peak 2, BS90-Sel1 only (additive model)**

| **Parameter Estimates** | | | | | | |
| --- | --- | --- | --- | --- | --- | --- |
| **Parameter** | **Estimate** | **Standard Error** | **Z** | **p-Value** | **95% Confidence Interval** | |
|  |  |  |  |  | **Lower** | **Upper** |
| Constant | -1.587 | 0.324 | -4.892 | 0.000 | -2.222 | -0.951 |
| Peak2 genotype | 0.857 | 0.324 | 2.646 | 0.008 | 0.222 | 1.493 |

| **Odds Ratio Estimates** | | | | |
| --- | --- | --- | --- | --- |
| **Parameter** | **Odds Ratio** | **Standard Error** | **95% Confidence Interval** | |
|  |  |  | **Lower** | **Upper** |
| Peak2 genotype | 2.357 | 0.764 | 1.249 | 4.449 |
